# Turkish Adaptation of the Screening Questionnaire for Intermittent Explosive Disorder

**DOI:** 10.31083/AP39496

**Published:** 2025-04-03

**Authors:** Sinem Çetin Demirtaş, Lut Tamam, Mehmet Emin Demirkol, Caner Yeşiloğlu

**Affiliations:** ^1^Psychiatry Clinic, Adana 5 Ocak State Hospital, 01100 Adana, Türkiye; ^2^Department of Psychiatry, Cukurova University Faculty of Medicine, 01380 Adana, Türkiye

**Keywords:** intermittent explosive disorder, reliability, scale adaptation, validity

## Abstract

**Background:**

Intermittent Explosive Disorder (IED) is an impulse-control disorder characterized by the inability to control emotions and behaviors, resulting in behaviors that violate social norms and the rights of others. The IED Screening Questionnaire (IED-SQ) is a valuable tool that can quickly identify the presence of IED in adults by Diagnostic and Statistical Manual of Mental Disorders-5 (DSM-5) criteria. This study evaluated a form of the IED-SQ that had been translated into Turkish, and investigated the reliability and validity of the Turkish version of the IED-SQ.

**Methods:**

Seventy-one adult IED patients and 68 adult controls participated. The Barratt Impulsivity Scale (BIS-11), Minnesota Impulse Control Disorder Interview Scale (MIDI), Buss Perry Aggression Questionnaire (BPAQ), Symptom Checklist 90-Revised (SCL-90-R), Wender Utah Rating Scale (WURS), and IED-SQ were administered to the participants.

**Results:**

The Cronbach’s α coefficient of the IED-SQ was 0.74. The subscales of the BPAQ, including physical aggression, verbal aggression, hostility, and anger, along with the attention and non-planning impulsivity subscales of the BIS-11, were incorporated into the multivariate analysis to identify factors independently associated with the IED-SQ. According to the model, the correct classification percentage was found to be 95%.

**Conclusions:**

This study demonstrated that the Turkish version of the IED-SQ is valid and reliable and can be used in clinical practices to identify the presence of IED.

## Main Points

1. Our study shows that the Turkish version of the IED-SQ scale is a valid and reliable measurement tool.

2. Our study represents the initial adaptation of the IED-SQ.

3. Our study revealed that the IED-SQ scale can be used in clinical practices to identify the presence of IED.

## 1. Introduction

Intermittent Explosive Disorder (IED) is an impulse-control disorder characterized by recurrent episodes of failure to resist impulses that result in severe acts of aggression or harm to others’ property [1]. IED was initially described by Esquirol in 1838 as “impulsive monomania”, and referred to as “partial madness” [2]. IED, originally termed “Passive-Aggressive Personality-Aggressive Type” in the Diagnostic and Statistical Manual of Mental Disorders-I (DSM-I), and “Explosive Personality” in the DSM-II, was referred to as IED in the DSM-III and thereafter. In the DSM-5, IED is classified under the category of “Disruptive, Impulse-Control, and Conduct Disorders”, alongside diagnoses like pyromania, kleptomania, and trichotillomania [3].

Although impulsive, aggressive behaviors and anger outbursts are commonly observed in society, the diagnosis of IED is often overlooked in psychiatric clinics. This low incidence of diagnosis frequently stems from shortcomings in the DSM criteria, and it has been argued that a more precise accounting of the prevalence of true IED in the community could be determined by revising the criteria. Coccaro developed “Research Criteria” (IED-RC) in an attempt to rectify the situation [4, 5]. Coccaro *et al*. [6] determined that the use of the IED-RC diagnostic criteria produced a highly reliable (0.87) level of diagnosis of lifelong IED. Cases diagnosed using IED-RC displayed higher levels of aggression, impulsivity, and impairment in functioning than did those diagnosed with Diagnostic and Statistical Manual of Mental Disorders, Fourth Edition, Text Revision (DSM-IV-TR) diagnostic criteria [4, 6]. The changes in the DSM-5 were primarily based on IED-RC [4].

IED patients struggle to restrain impulses that lead to verbal or physical outbursts. IED patients’ aggressive behaviors are often impulsive and disproportionate to provocation. Individuals with IED may experience intense distress, psychosocial impairment, and self-harm tendencies [7]. Individuals with IED frequently exhibit comorbid psychiatric disorders. Although the comorbidity of Attention-Deficit/Hyperactivity Disorder (ADHD) and IED has not been extensively examined in previous studies, it is not surprising to see them co-occurring, given that both disorders involve issues with impulse control [3, 8].

Hypotheses regarding the etiology of impulsive aggression and IED can be categorized into two main themes. The first involves adverse early childhood experiences that may affect the development of crucial attributes such as self-control, impulse control, deferred gratification, and the ability to cope with frustration, all of which are central to suppressing impulsive aggression. The second theme suggests that impulsive aggression is associated with imbalances in brain function related to behavioral arousal and inhibition; this perspective has gained strength over the past two decades with the support of numerous research findings [9].

Epidemiological studies of IED suggest that this diagnosis may be more common than previously thought [10, 11, 12]. Felthous *et al*. [13] diagnosed 3.4% of 443 male volunteers with complaints of violence and aggression as IED. Coccaro *et al*. [14] identified a lifetime prevalence of 3.95% for IED among 253 individuals in Baltimore, but when the same sample was evaluated using Intermittent Explosive Disorder - Research Criteria (IED-RC), the prevalence increased to 5.14%. Gelegen and Tamam found the lifetime and annual prevalence rates of IED in 406 patients seeking outpatient services for the first time to be 16.7% and 11.3%, respectively, according to DSM-5 criteria [5].

IED can potentially lead to significant public health issues, as it is a treatable, widespread behavioral disorder. Individuals with IED may not actively seek treatment, so recognizing this diagnosis is of paramount importance. Moreover, the need for a scale that can facilitate diagnosis is evident [5, 15]. Intermittent Explosive Disorder Screening Questionnaire (IED-SQ) is considered a potentially valuable screening tool for identifying the presence of IED in adults [16]. The present study was designed to assess the validity and reliability of a Turkish adaptation of the IED-SQ that was developed by Coccaro.

## 2. Materials and Methods

### 2.1 Translation Process

Permission for the study was obtained from Coccaro via e-mail. After independent translation into Turkish by two psychiatrists, the scale was translated back into English, and linguists assessed its linguistic equivalence. It was deemed that the translation was appropriate.

### 2.2 Study Setting and Subjects

Patients who were admitted with anger control problems to the Çukurova University Faculty of Medicine Psychiatry Outpatient Clinic and received a diagnosis of IED, according to DSM-5 criteria, between March 7, 2021 and September 7, 2021, were included. According to the results of the power analysis conducted with the G Power 3.1 program, 80% power with a 0.05 error margin using Cohen’s formula, the study planned to include 130 adult patients, 65 each in the patient and control groups. A patient group was formed consisting of 95 IED patients, and a control group was formed consisting of 95 people who lived in the same environment as the IED patients and who had similar sociodemographic characteristics. The first author conducted a psychiatric examination of the participants based on DSM-5 criteria. Two individuals from the patient group were excluded from the study due to mental retardation, one was excluded due to dementia, three due to manic episodes, nine due to psychotic episodes, and one due to a major depressive episode. Those individuals were excluded because their conditions were deemed to impede the proper continuation of the interview. Physical disorders such as endocrine diseases and neurodegenerative diseases were also considered as exclusion criteria because they may cause anger-like symptoms, as in IED. After conducting a mental examination and confirming the diagnosis of IED according to DSM-5 criteria, we administered the Sociodemographic Data Form, Wender Utah Rating Scale (WURS), Barratt Impulsiveness Scale (BIS-11), Minnesota Impulse Control Disorder Interview (MIDI), Buss Perry Aggression Questionnaire (BPAQ), Symptom Checklist-90-Revised (SCL-90-R), and the Intermittent Explosive Disorder Screening Questionnaire (IED-SQ) to the IED patients and the controls. These scales were also selected in addition to the IED-SQ because they had been used in similar studies and because the individuals considered to be the patient group exhibited symptoms that these scales could assess. Despite being conducted in different populations (university students, 10th-grade students, psychiatric outpatients), different countries (Turkiye, Iran, USA), and with varying sample sizes, these studies have consistently observed that impulsivity, aggression, comorbid psychiatric symptoms, and ADHD-like symptoms are more prevalent in patients with IED compared with healthy individuals [5, 16, 17, 18, 19].

Ethical approval for our study was obtained from the Non-Interventional Clinical Research Ethics Committee of Çukurova University Faculty of Medicine on March 5, 2021 (decision number 33). Participants were informed about the process during the interviews, and written informed consent was obtained. Participants initially completed self-report questionnaires. The remaining data were gathered through face-to-face interviews by the first author. Twenty-seven participants in the control group and nine in the IED group were excluded due to missing data. The study was therefore continued with 139 participants, comprising 71 patients and 68 control subjects.

### 2.3 Measures and Procedure

#### 2.3.1 Intermittent Explosive Disorder Screening Questionnaire

The development of a screening approach for diagnosing IED was initiated with an examination of the association between IED and data obtained from the Life History of Aggression (LHA) assessment because research suggests that lifelong patterns of aggressive behavior may be linked to impulsive disorders such as IED. We thought that including items from the LHA in the IED-SQ might facilitate the accuracy of diagnosis and understanding of general levels of aggression [16, 20]. The LHA is a widely used measure designed to evaluate an individual’s life history of overt aggressive behavior. Administered as a semi-structured interview, this assessment comprises three subscales: aggression, consequences, and self-directed aggression; the latter encompassing suicidal attempts. Due to the good psychometric properties for screening DSM-5 IED, only the first two subscales of the LHA are preferred [16].

During scale development, a new set of seven items was created to be added to the five LHA Aggression items, in order to identify DSM-5 IED in adult research participants. These seven items included: (1) frequency of verbal/non-destructive aggression (A1) and moderation of these behaviors (E); (2) frequency of destructive/aggressive aggression (A2) and moderation of these behaviors (E); (3) proportionality of aggressive responses to provocation (B); (4) impulsive or planned nature of aggressive outbursts (C); (5) distress and/or impairment resulting from aggressive outbursts (D); (6) exclusionary factors related to aggression involving the presence of other psychiatric disorders (F1); and (7) exclusionary factors related to the behavioral impact of medication or substances of abuse on aggressive outbursts (F2). We observed that aggressive individuals had difficulty distinguishing between appropriate and disproportionate behavioral responses to social threats and to the effects of other psychiatric disorders. Accordingly, items related to these two criteria (B and F1) were removed.

The internal consistency for these subscales was found to be 0.87 for Aggression, and 0.48 for Self-Directed Aggression. We observed that due to the consistent psychometric properties of the first two subscales, their potential as a screening approach for DSM-5 IED could be evaluated. The original research program identified a high specificity (90.5%) but low sensitivity (61.0%) for DSM-5 IED diagnosis. A closer examination revealed that the low sensitivity was attributable to the fact that the LHA consequence subscale solely assesses the functional impairment aspect of IED, omitting other criteria of DSM-5 IED. Consequently, five questions were developed and added to the existing five LHA Aggression items, in order to identify DSM-5 IED in adult research participants. A lifetime diagnosis of DSM-5 IED was considered to be present when the respondent scored ≥12 on the first five items and provided affirmative answers on items 1a, 2, 3, 4a/4b, and 5a/5b [16].

#### 2.3.2 Sociodemographic Data Form

The Sociodemographic Data Form tabulated participants’ age, gender, education, socioeconomic status, history of childhood trauma, psychiatric diagnoses and treatment, the presence of anger outbursts involving damage to property, verbal aggression, interpersonal violence or threats, lifetime suicide attempts or self-injurious behavior, and a tendency towards violence within the family. This form was completed through one-on-one interviews conducted by the first author.

#### 2.3.3 Semptom Checklist-90-Revised (SCL-90)

The Semptom Checklist-90-Revised (SCL-90) is a symptom-screening questionnaire that assesses psychological symptoms and has an adequate interrater reliability of 0.65–0.8. It is structured to evaluate 10 distinct symptom dimensions, including somatization, depression, anxiety, obsessions-compulsions, hostility, paranoid ideation, psychoticism, interpersonal sensitivity, paranoid anxiety, and an additional scale [21]. The reliability of the SCL-90-R is high, with Cronbach’s α ranging from 0.665 to 0.848. Reliability of the total items was high with a reported α of 0.966 [21]. In our study, the Cronbach’s α value of SCL-90 was found to be 0.98.

#### 2.3.4 Buss-Perry Aggression Questionnaire (BPAQ)

The Buss-Perry Aggression Questionnaire (BPAQ) is used to assess an individual’s aggression, measuring both the dimensions of anger and hostility, and physical and verbal aggression. The BPAQ comprises 28 items. Physical aggression is assessed in items 1–6 and 8–9, verbal aggression in items 10–14, anger in items 15–21, and hostility in items 22–29. It uses a 5-point Likert-type assessment scale. The internal consistency coefficient (Cronbach’s α) was reported to be 0.89 for Factor 1 “Physical Aggression”, 0.84 for Factor 2 “Hostility”, 0.82 for Factor 3, 0.59 for Factor 4, and 0.93 for the total scale score [22]. In our study, the Cronbach’s α value of BPAQ was found to be 0.95.

#### 2.3.5 Barratt Impulsiveness Scale-11 (BIS-11)

The Barratt Impulsiveness Scale-11 (BIS-11) is a self-report questionnaire used to assess impulsivity. The scale consists of 30 items and includes three sub-scales: attention impulsivity, motor impulsivity, and non-planning. The higher the total BIS-11 score, the greater the level of impulsivity exhibited by the individual [23]. Cronbach’s α was reported to be 0.85 for the total scale score [24]. In our study, the Cronbach’s α of BIS-11 was found to be 0.91.

#### 2.3.6 Minnesota Impulse Control Disorder Interview (MIDI)

The Minnesota Impulse Control Disorder Interview (MIDI) comprises 36 items. It assesses impulse control disorders according to DSM-IV criteria, including IED, pathological gambling, trichotillomania, pyromania, kleptomania, compulsive buying, compulsive sexual behavior, skin picking, and compulsive exercise [25]. In the present study, the Cronbach’s α value of MIDI was 0.93.

#### 2.3.7 Wender Utah Rating Scale

The Wender Utah Rating Scale (WURS) was developed to assess childhood symptoms related to Adult Attention-Deficit/Hyperactivity Disorder (ADHD). The total score of the scale is computed by summing the scores across all items. It has a cut-off value of 36 [26]. Cronbach’s α was reported to be 0.93 for the total scale score [27]. In our study, the Cronbach’s α value of WURS was found to be 0.95.

### 2.4 Statistical Analysis

Assuming that skewness and kurtosis values fall within the range of –1.5 to +1.5, the sample size for the study ranged between 50 and 300, and the *z*-score was in the range of –3.29 to 3.29, we posited that the variable distributions exhibited normality based on histogram observations [3, 28]. Independent samples *t*-tests were used to analyze datasets exhibiting normal distribution and the Mann–Whitney U test was used for non-normally distributed data. Yates’ statistic was applied when expected observation counts ranged from 5 to 25; otherwise, the Chi-squared test was used. Descriptive statistics were presented as either the mean (standard deviation) or median (interquartile range), contingent upon the data distribution. Statistical significance was considered to have existed when *p*-values were ≤0.05.

In our study, logistic regression analysis was applied, utilizing the Hosmer-Lemeshow test, the omnibus test, the backward-stepwise model, Nagelkerke R Squared, and Cox & Snell R Squared during the process.

Given the significant differences between the patient and control groups in terms of age, marital status, and place of residence, and considering that the symptom profile of the patients aligned with the clinical presentation of individuals with anger management issues and impulsivity, the BPAQ (subscales: physical aggression, verbal aggression, hostility, and anger) and BIS-11 (subscales: attention, motor, and non-planning) were included in the multivariate analysis to identify the factors independently associated with the IED-SQ. Concurrent validity was demonstrated by using the MIDI for the IED.

## 3. Results

We found that IED is more frequently diagnosed in men, young people, and individuals living in city centers rather than small towns. No association was found between IED and gender, marital status, educational level, or socioeconomic status (Table 1).

**Table 1.** **Comparison of the Sociodemographic Distributions of Individuals in Patient and Control Groups**.

Variable		Patient	Control	Statistic
Gender				0.53
	Female	20 (28.2)	15 (22.1)	
	Male	51 (71.8)	53 (77.9)	
Marital status				0.42
	Married	34 (47.9)	40 (58.7)	
	Single	37 (52.1)	28 (41.2)	
Education				0.97
	Elementary school	17 (23.9)	17 (25)	
	High school	36 (50.7)	35 (51.5)	
	University	18 (25.4)	16 (23.5)	
Employment status				*p* < 0.001*
	Unemployed	22 (30.9)	5 (7.3)	
	Employed	49 (69)	63 (92.6)	
Socioeconomic status				0.15
	Lower	13 (18.3)	9 (13.2)	
	Middle	51 (71.8)	57 (83.8)	
	Upper	7 (9.9)	2 (2.9)	
Place of residence				*p* < 0.001*
	City center	39 (54.9)	58 (85.3)	
	Small town	32 (45)	10 (14.7)	
Age (years)		35.32 ± 10.33	40.32 ± 10.40	0.01*

*The *p*-value indicates statistical significance at the 0.05 level.

BPAQ, BIS-11, and SCL-90-R total scores and subscale averages were higher in the patient group than in the control group (Table 2). There was a significant relationship between the groups and the WURS score, with more individuals testing positive in the patient group than in the control group. According to the MIDI-IED scale, no IED individuals were in the control group, whereas 84.5% of the patient group tested positive for IED. Using the IED-SQ, 87.3% of the patient group tested positive, with no positive individuals in the control group.

**Table 2.** **Comparison of Scale Scores between Patient and Control Groups**.

Scale		Patient	Control	Statistic
BPAQ Total, mean ± SD		102 ± 25.05	53.33 ± 14.25	*p* < 0.001*
BPAQ Physical Aggression, mean ± SD		28.75 ± 8.42	12.42 ± 3.56	*p* < 0.001*
BPAQ Verbal Aggression, mean ± SD		18.28 ± 8.58	12.31 ± 3.48	*p* < 0.001*
BPAQ Anger, mean ± SD		27.54 ± 6.68	14.18 ± 5.37	*p* < 0.001*
BPAQ Hostility, mean ± SD		27.44 ± 7.71	14.42 ± 4.87	*p* < 0.001*
BIS-11 Total, mean ± SD		78.65 ± 16.29	55.90 ± 7.72	*p* < 0.001*
BIS-11 Attention Impulsivity, mean ± SD		21.10 ± 4.55	14.13 ± 2.94	*p* < 0.001*
BIS-11 Motor Impulsivity, mean ± SD		25.18 ± 6.59	17.62 ± 2.89	*p* < 0.001*
BIS-11 Non-planning, median (IQR)		33 (26–38)	24 (22–26)	*p* < 0.001*
SCL-90 General Symptom Index, mean ± SD		1.62 ± 0.75	0.66 ± 0.51	*p* < 0.001*
Somatization, median (IQR)		12 (6.75–21)	7 (3–12)	*p* < 0.001*
Obsessive-compulsive, mean ± SD		15.41 ± 8.92	10.71 ± 7.74	*p* < 0.001*
Interpersonal sensibility, mean ± SD		15.5 ± 8.36	6.73 ± 6.01	*p* < 0.001*
Depression, mean ± SD		23.19 ± 11.31	10.03 ± 7.13	*p* < 0.001*
Anxiety, mean ± SD		16.64 ± 9.96	4.59 ± 4.08	*p* < 0.001*
Anger-Hostility, median (IQR)		17.5 (9–20)	1 (0–3)	*p* < 0.001*
Phobic-Anxiety, mean ± SD		8.06 ± 6.26	2.18 ± 2.13	*p* < 0.001*
Additional Items, mean ± SD		11.40 ± 6.65	5.56 ± 5.38	*p* < 0.001*
WURS, n (%)				*p* < 0.001*
	Patient	66 (93)	46 (67.6)	
	Control	5 (7)	22 (32.4)	
MIDI-IED, n (%)				*p* < 0.001*
	Patient	60 (84.5)	0	
	Control	11 (15.5)	68 (100)	
IED-SQ, n (%)				*p* < 0.001*
	Patient	62 (87.3)	0	
	Control	9 (12.7)	68 (100)	

BPAQ, Buss Perry Aggression Questionnaire; BIS-11, Barratt Impulsiveness Scale; IED-SQ, Intermittent Explosive Disorder Screen Questionnaire; MIDI-IED, Minnesota Impulsivity Disorders Interview; WURS, Wender Utah Rating Scale; SD, Standard deviation; SCL-90, Semptom Checklist-90-Revised. *The *p*-value indicates statistical significance at the 0.05 level.

Based on the Hosmer-Lemeshow test, the model fits well (*p* = 0.83), with Nagelkerke R-Squared at 0.81 and Cox & Snell R-Squared at 0.60. The independent variables accounted for 81% of variance in the IED-SQ variable (Table 3).

**Table 3.** **Model Summary and Hosmer Lemeshow Test of the IED-SQ**.

Model Summary		
–2 Log Likelihood	Cox & Snell R Squared	Nagelkerke R Squared
61.87	0.60	0.81
Hosmer and Lemeshow Test		
Chi-squared	df	Sig.
4.26	8	0.83

Sig, Significant at ≤0.05; df, degrees of freedom.

The subscales of the BPAQ, including physical aggression, verbal aggression, hostility, and anger, along with the attention impulsivity, motor impulsivity, and non-planning subscales of the BIS-11, and the variables age, marital status, and place of residence were incorporated into the multivariate analysis to identify factors independently associated with the IED-SQ. The analysis only revealed that the physical and verbal aggression subscales of the BPAQ and the attention subscale of the BIS-11 were independently associated with IED-SQ (Tables 4,5).

**Table 4.** **Comparison of the effective factors on the IED-SQ**.

	B	S.E.	Wald	df	Sig.	Exp(B)
BPAQ Physical Aggression	–0.382	0.093	17.028	1	0.000	0.682
BPAQ Verbal Aggression	0.154	0.064	5.703	1	0.017	1.166
BIS-11 Attention Impulsivity	–0.398	0.135	8.643	1	0.003	0.671

Sig, *p* value; B, coefficient; S.E., standard error; df, degrees of freedom; Wald, Wald statistic; Exp(B), odds ratio.

**Table 5.** **Multivariate regression analysis summary**.

Parameters	Odds Ratio	95% Confidence Interval		*p*-value
BPAQ Physical Aggression	0.682	0.569	0.818	<0.001
BPAQ Verbal Aggression	1.166	1.028	1.323	0.017
BIS-11 Attention Impulsivity	0.671	0.515	0.876	0.003

A backward-stepwise model was used and the last step (step 5) is shown in Table 5. The Omnibus test for this model had a *p*-value of <0.001.

Our study revealed that the Cronbach’s α values for the scales were as follows: 0.95 for WURS; 0.95 for BPAQ; 0.91 for BIS-11; 0.98 for SCL-90-R; 0.93 for MIDI; and 0.74 for IED-SQ.

## 4. Discussion

The investigation of human aggression has been hindered for years by the need for more reliable and valid diagnostic criteria for individuals exhibiting impulsive aggressive behavior. IED, which belongs to this behavioral category, needs more and better psychometric measures [11]. Our study’s most significant outcome was the demonstration of the validity and reliability of the Turkish version of IED-SQ. It was observed in the original study by Coccaro that the IED-SQ demonstrated high specificity (90.5%) but low sensitivity (61.0%) [16]. However, our study found a specificity of 100% and a sensitivity of 87.3%, with only 12% of the patient group being overlooked. This may be attributed to including LHA in the DSM-5 diagnostic criteria, which requires more criteria to be met. The combined sensitivity and specificity percentages exceeding 170 suggest that the tests are clinically sound, thus supporting the validity of the IED-SQ as a screening tool [29].

In the original study by Coccaro *et al*. [16], the internal consistency coefficient of the IED-SQ was 0.80. Barra *et al*. [30] found that the IED-SQ internal consistency coefficient was 0.79 in 161 young adults; Gharibpour and Akbari [31] found an α of 0.85 in 54 prisoners; and Sheikhi Gerakoui [17] found it to be 0.72 in 665 10th-grade students. In the present study, the Cronbach’s α coefficient of the Turkish version of IED-SQ was 0.74, indicating that the internal consistency was adequate [32].

Previous studies have shown that IED is more common in men than in women [6, 33, 34]. We also observed a 2.5 times higher incidence in men compared with women. Contrary to a previous study in our country, we found that most IED patients (54.9%) reside in city centers [5]. It is worth considering if urban living conditions trigger impulsivity and aggression. Studies have shown that unemployment is a risk factor for IED [5, 35]. In our study, the higher rate of unemployment in the patient group compared with the control group supports this finding. Our study found no relationship between marital status, education level, or socioeconomic status and the patient or control groups. The disease is more prevalent in younger individuals (<35–40 years), as consistent with previous studies [15, 34].

Our study showed that significant differences exist in the IED-SQ scores of the patient and control groups in the BPAQ, BIS-11, and SCL-90-R total and subscale scores, as well as in the WURS and MIDI results. These findings are consistent with those reported in existing literature, and support previous studies by Coccaro, Sheikhi Gerakoui, and Kulper, *et al*. [17, 19, 31, 36, 37, 38], indicating that individuals diagnosed with IED exhibit higher levels of psychopathology, aggression, and impulsivity.

In reviewing the current literature, we observed that individuals diagnosed with IED are likely to experience depression and anxiety symptoms at least four times more frequently than the general population, which is consistent with data from studies conducted by Kessler *et al*. [11] and from other similar community-based investigations [39, 40]. In the present study, consistent with these findings, individuals diagnosed with IED were found to have 2.5 to 3 times higher rates of depressive and anxiety symptoms than those without the diagnosis. In our study, anxiety and depression were evaluated not as diseases but as symptoms, aiming to avoid confounded effects. However, from another perspective, this approach can also be considered a limitation.

McElroy *et al*. [33] found that obsessive-compulsive symptoms were reported in 22% of individuals with IED. Our results, in line with current literature, showed that 22.5% of patients diagnosed with IED, based on the IED-SQ, reported symptoms of obsession and compulsion. Additionally, ADHD is considerably more prevalent in IED patients (20%) than in the general population [5, 41, 42]. In our study, supporting other studies, approximately 90% of the patient group diagnosed with IED, according to the IED-SQ, exhibited symptoms similar to ADHD as indicated by the WURS results.

In both previous studies and in our own study, the absence of investigation into the relationship between types of obsessive-compulsive symptoms and types of ADHD underscores the necessity for research in this area.

When the results of the logistic regression analysis were evaluated, the variables contributing significantly to the model were the physical and verbal aggression subscales and the attention subscale. A literature review showed that the IED-SQ, BPAQ, and BIS-11, and their subscales, have been studied together in most studies, and almost all of them showed a high correlation with IED, as we found in our study. The frequent occurrence of anger and impulsivity in patients with IED may have contributed to this result [5, 16, 43].

Among individuals classified as IED patients according to the Turkish version of the IED-SQ, 85.5% were also classified as IED patients according to the MIDI-IED, whereas among those classified as healthy, according to IED-SQ, 90.9% were classified as non-IED according to the MIDI-IED. Our results demonstrated that the IED-SQ has appropriate equivalent validity. The absence of equivalent validity studies for the IED-SQ in the literature suggests that our study could guide future research.

One of the strengths of the present study is that it is one of the few focusing on DSM-5 criteria-based IED in Turkey and is the first study to apply the IED-SQ to a Turkish sample. Participants’ diagnoses were standardized and conducted through structured clinical interviews by a single researcher. To the best of our knowledge, this is the only study investigating the validity and reliability of the IED-SQ in a language other than English.

One of the limitations of our study is the cross-sectional design, which weakens our ability to establish causality. Other limitations are the predominantly self-reporting nature of the scales used, the reliance on entirely self-reported socioeconomic status of participants and their families, psychiatric histories, and the acceptance of obsessions and compulsions, anxiety, and depression as symptoms rather than illnesses. Additionally, the study lacks retest measurements and did not involve inter-rater reliability assessments. Future studies that (a) consider depression and anxiety, which we treated as symptoms, as comorbid conditions; (b) involve larger sample sizes; and (c) include re-test and inter-rater reliability measurements, may help to obtain more comprehensive results.

## 5. Conclusions

Our study shows that the Turkish version of the IED-SQ scale is a valid and reliable measurement tool with adequate internal consistency, convergent validity, and equivalent validity. As far as we know, this study represents the first attempt to adapt the IED-SQ to another language. We believe that the Turkish version of the IED-SQ will help to identify Turkish-speaking IED patients more quickly.

## Availability of Data and Materials

The data presenting in this study are available on request from the corresponding author.
